# Aberrations in Cell Signaling Quantified in Diabetic Murine Globes after Injury

**DOI:** 10.3390/cells13010026

**Published:** 2023-12-21

**Authors:** Nicholas A. Azzari, Kristen L. Segars, Srikar Rapaka, Landon Kushimi, Celeste B. Rich, Vickery Trinkaus-Randall

**Affiliations:** 1 Department of Biochemistry and Cell Biology, Boston University Chobanian and Avedisian School of Medicine, 72 E. Concord St., Boston, MA 02118, USA; nazzari@bu.edu (N.A.A.); cbrich@bu.edu (C.B.R.); 2 Department of Pharmacology, Physiology and Biophysics, Boston University Chobanian and Avedisian School of Medicine, 72 E. Concord St., Boston, MA 02118, USA; ksegars@bu.edu; 3 Department of Medicine, Boston University Chobanian and Avedisian School of Medicine, 72 E. Concord St., Boston, MA 02118, USA; sjrapaka@bu.edu; 4 Department of Computer Science, Center for Computing and Data Sciences, Boston University, 665 Commonwealth Ave, Boston, MA 02115, USA; lsk1801@bu.edu; 5 Department of Ophthalmology, Boston University Chobanian and Avedisian School of Medicine, 72 E. Concord St., Boston, MA 02118, USA

**Keywords:** cornea, diabetes, cell signaling, wound healing, live cell imaging

## Abstract

The corneal epithelium is an avascular structure that has a unique wound healing mechanism, which allows for rapid wound closure without compromising vision. This wound healing mechanism is attenuated in diabetic patients, resulting in poor clinical outcomes and recurrent non-healing erosion. We investigated changes in cellular calcium signaling activity during the wound response in murine diabetic tissue using live cell imaging from both ex vivo and in vitro models. The calcium signaling propagation in diabetic cells was significantly decreased and displayed altered patterns compared to non-diabetic controls. Diabetic cells and tissue display distinct expression of the purinergic receptor, P2X7, which mediates the wound healing response. We speculate that alterations in P2X7 expression, interactions with other proteins, and calcium signaling activity significantly impact the wound healing response. This may explain aberrations in the diabetic wound response.

## 1. Introduction

Understanding the connection between changes in cellular signaling profiles and reduced capacity for wound healing with age and disease is of broad interest to many fields, including avascular wound healing in the cornea. Our laboratory has developed methodologies to image intact globes that allow us to examine how cells communicate with each other in their proper 3-D environment [1,2]. The corneal epithelium is the outermost structure of the eye and has a protective role, as it is subjected to a wide range of environmental stimuli and acts as a barrier to prevent damage to underlying structures [3]. In corneal wound healing, epithelial cells move as a contiguous sheet. The motility is caused by protrusion of lamellipodia at the leading edge of the wound, requiring a continual assembly and disassembly of focal adhesions that occur during extension, retraction, and maturation of nascent adhesions [3,4,5,6,7,8,9,10]. Comparing the signaling profiles recorded from heathy and diabetic mouse models will give insight regarding why wound healing may be impaired.

As diabetic corneas are susceptible to epithelial defects arising from delayed or impaired wound healing, our study of the cellular signaling response in an intact diabetic globe will be informative [11,12,13]. Previously, we demonstrated that both the epithelium and stroma of healthy murine eyes become significantly stiffer with increased age, while the basement membrane does not change [1,13,14,15,16,17,18]. In contrast, there is a decrease in both stiffness and sensory nerve density in obese mice [11,14]. It is not understood how these changes affect cell–cell communication.

The goal of this manuscript is to examine the differences in post-injury calcium signaling in diabetic murine globes and in primary diabetic cultures compared to controls to determine potential causes of these changes. We hypothesize that there are decreases in the calcium signaling propagation and in the pattern of signaling between cells after injury in the diabetic corneal epithelium in both in vitro and ex vivo. We used live cell imaging of both in vitro and ex vivo control and diabetic models and quantification of the calcium signaling response after injury to test the hypothesis. In addition, we evaluated changes in P2X7 expression in control and diabetic models.

We have found that there are major changes in cell–cell communication between corneal epithelial cells during the wound response, and in interactions between proteins known to be involved in the wound response from prior studies on obese, prediabetic, and diabetic models [1,19,20,21]. These findings characterize some of the changes in the wound healing response in diabetic tissue and present further opportunities for future studies to advance our understanding of changes in corneal wound healing response with disease and age.

## 2. Materials and Methods

### 2.1. Animals

C57BL/6J, BALB/cJ, and NONcNZO10/LtJ mice aged 9–12 weeks were purchased from The Jackson Laboratory (Bar Harbor, ME, USA). The NONcNZO10/LtJ mice represent a Type 2 diabetic model. The research protocol conformed to the standards of the Association for Research in Vision and Ophthalmology for the Use of Animals in Ophthalmic Care and Vision Research and the Boston University IACUC protocol.

### 2.2. Cell Culture

Human corneal limbal epithelial (HCLE) cells were telomerase-immortalized and were a gift from Dr. Gipson (Schepens Eye Research Institute/Mass. Eye and Ear, Boston, MA, USA) and were verified at Johns Hopkins DNA Services (Baltimore, MD, USA). Cells were maintained in Keratinocyte Serum-Free Medium (KSFM) with the following growth supplements (25 μg/mL bovine pituitary extract, 0.02 nM EGF, Penicillin/Streptomycin (100 U/mL/100 ug/mL, respectively), and 0.3 mM CaCl_2_) [22,23,24].

Human primary corneal epithelial (HPri) (H-6048) and human primary diabetic kidney epithelial (HDia) (HD2-6034) cells were purchased from Cell Biologics Inc. (Chicago, IL, USA) and maintained in Keratinocyte Serum-Free Medium (KSFM) with the following growth supplements (25-μg/mL bovine pituitary extract, 0.02 nM EGF, Penicillin/Streptomycin (100 U/mL/100 ug/mL, respectively), 0.3 mM CaCl_2_, Insulin-Transferrin-Sodium Selenite, Hydrocortisone, Antimycotic, and fetal bovine serum).

Murine primary (MsPri) (db-6048) and primary Diabetic (MsDia) (MD-6048) corneal epithelial cells were purchased from Cell Biologics Inc. (Chicago, IL, USA) and maintained in Keratinocyte Serum-Free Medium (KSFM) with the following growth supplements (0.02 nM EGF, Penicillin/Streptomycin (100 U/mL/100 ug/mL, respectively), 0.3 mM CaCl_2_, Insulin-Transferrin-Sodium Selenite, Antimycotic, and fetal bovine serum).

For live cell imaging assays, the cells were plated on p35 MatTeK glass-bottom dishes (MatTek Corporation, Ashland, MA, USA) and grown to confluence; 12 to 24 h prior to live cell imaging, medium was changed to unsupplemented KSFM as described [19].

### 2.3. Live Cell Imaging

Live cell imaging and calcium mobilization experiments were performed as described previously [1,19]. Cells were preincubated with ARL 67156 trisodium salt (ARL) (Sigma-Aldrich (St. Louis, MO, USA)) (10, 20, 50 nM) [25,26], Fluo-4AM (Thermo Fisher, Waltham, MA, USA) (1:100) and the counterstain SirActin (Cytoskeleton, Inc., Denver, CO, USA) (1:1000) for 20 min at 37 °C. Images were collected every 3 s for 45 min on the Zeiss Axiovert LSM 880 confocal microscope (Zeiss, Thornwood, NY, USA) utilizing the 20× air objective. Scratch wounds or agonist stimulation by ATP, 2,3-O-(4-benzoylbenzoyl)-ATP (BzATP) (Sigma-Aldrich (St. Louis, MO, USA)), or ATP (Sigma-Aldrich (St. Louis, MO, USA)) were performed after 100 frames (5 min). All injuries were made using a 25-gauge needle.

### 2.4. Proximity Ligation Assay (PLA)

Detection of P2X7 and Pannexin-1 proteins by Proximity Ligation Assay (PLA) was performed as described previously using anti-pannexin1 monoclonal mouse antibody and anti-P2X7 polyclonal rabbit antibody [20] (Alomone Labs (Jerusalem, Israel)). Detection of antibodies was performed using Duolink^®^ In Situ PLA^®^ Probe anti-mouse MINUS (DUO92004) and donkey anti-rabbit PLUS (DUO92002) (Sigma-Aldrich, St. Louis, MO, USA) as described previously [20]. Tissue sections near and far from the wound were imaged using the Zeiss Axiovert LSM 700 (Zeiss, Thornwood, NY, USA). Analysis was performed using FIJI/ImageJ 2.14 (NIH, Bethesda, MD, USA; http://imagej.nih.gov/ij/ (accessed on 27 September 2022)) and Cell Profiler (Broad institute, Cambridge, MA, USA) [20,27,28]. Statistical analysis was performed using ANOVA, Tukey Post hoc, and Student *t*-tests in Excel 16.16.27 (Microsoft Corporation (Redmond, WA, USA)).

### 2.5. SDS PAGE Electrophoresis

Cells were cultured for 72 h until confluent and lysed. Lysates were collected in a Tris/NaCl buffer containing 150 mM NaCl, 10 mM Tris, 1 mM EGTA, 1 mM EDTA, 0.5% NP-40, 2 mM PMSF, and 2 mM Na_3_VO_4_. The lysates were sonicated and centrifuged at 10,000 rpm and the supernatant was collected. Protein concentration was determined using a NanoDrop One (Thermo Fisher, Waltham, MA, USA). Sixty micrograms of protein from each lysate were subjected to SDS-PAGE (12%) and transferred to a PVDF membrane (Bio-Rad, Hercules, CA, USA).

Immunoblots were blocked in a 10 mM Tris buffer (TBST: 10 mM Tris, 100 mM NaCl, 0.1% Tween-20 pH 7.4) containing 0.5% BSA (Thermo Fisher, Waltham, MA, USA) and membranes were incubated with appropriate antibodies, washed, incubated with appropriate secondary antibodies, and rinsed with TBST. PVDF membranes were blocked in TBST 5% BSA blocking solution for 1 h prior to antibody incubations. PVDF membranes were incubated in Tris and 1% BSA blocking buffer containing the primary antibody (1:100) overnight at 4 °C. Anti-P2X7 polyclonal rabbit antibody (Cat. #APR-004 and APR-008) was purchased from Alomone Labs (Jerusalem, Israel). The Alexa Fluor-conjugated secondary antibody (Invitrogen, Carlsbad, CA, USA) was used at a dilution of 1:200 in 1% BSA blocking solution for 1 h at room temperature. Visualization was performed using a VersaDoc Imaging System (Bio-Rad, Hercules, CA, USA). Analysis was performed in FIJI/ ImageJ 2.14 (NIH, Bethesda, MD, USA; http://imagej.nih.gov/ij/ (accessed on 27 September 2022)).

### 2.6. Ex Vivo Live Cell Imaging

Live cell imaging experiments were performed on intact globes as described previously [1,2]. Mice were euthanized and intact globes were removed. Globes were preincubated with Fluo-4 AM(1:100) and counterstain CellMask™ Deep Red (Thermo Fisher, Waltham, MA, USA) (1:10,000). Puncture wounds were induced on the central cornea of the intact globes prior to imaging using a 25-gauge needle. Wounds have previously been demonstrated to have consistent size and depth [1]. Images were collected every 3 s for 60 min with the Zeiss Axiovert LSM 880 (Zeiss, Thornwood, NY, USA) confocal microscope utilizing the 20× air objective, time series, and z-stack experimental parameters for imaging.

### 2.7. Analysis

Imaging data from ex vivo murine globes was imported into MATLAB and stabilized using Non-Rigid Motion Correction (NoRMCorre) algorithms [29]. Cellular identification was performed using Region of Interest (RoI) selection in FIJI/ImageJ2.14 for ex vivo preparations and with MATLAB binary masking algorithms for in vitro preparations. Image analysis in FIJI/ImageJ 2.14 (NIH, Bethesda, MD, USA; http://imagej.nih.gov/ij/ (accessed on 27 September 2022)) and MATLAB (MATLAB, MathWorks, Inc., Natick, MA, USA) was performed to generate figures that display changes in intensity that are representative of calcium activity. The algorithms detect individual signaling events and propagation from cellular intensity and location data.

Topographical maps of signal intensity were created using Z-project in FIJI/ImageJ 2.14 and Zen Blue (Zeiss, Thornwood, NY, USA) to determine the location of cellular calcium signaling events. Processing time series data using maximum intensity revealed all locations where a signaling event occurred. Images were imported into Zen Blue to create 2.5D images where red/white spikes represent an intensity-shifted LUT scale.

## 3. Results

### 3.1. Initial Response Calcium Wave Comparison

**Figure 1:**
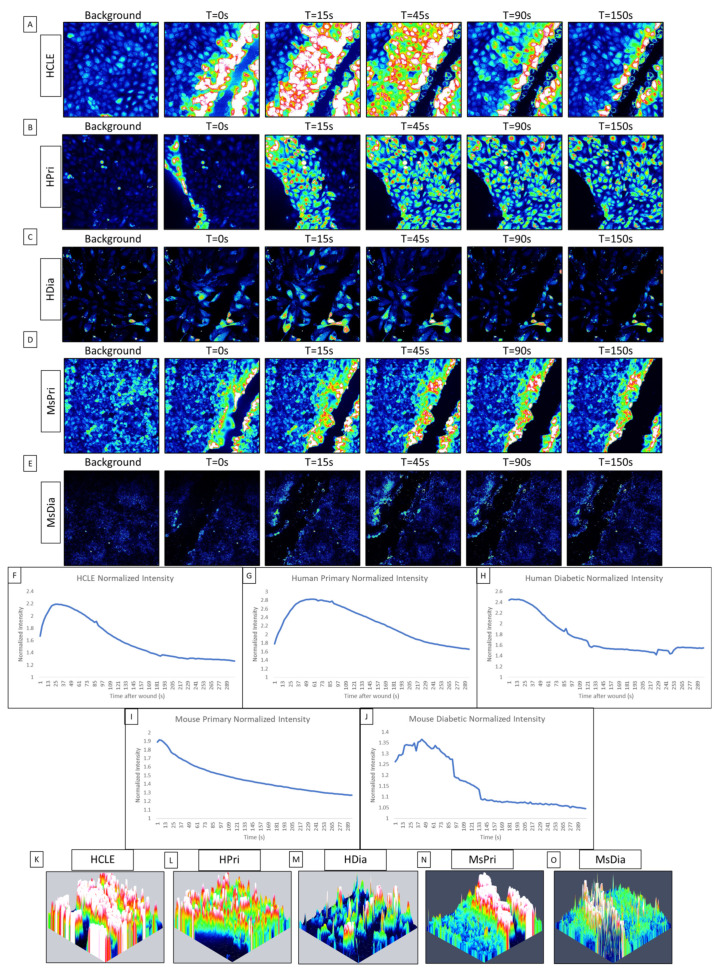
Comparison of initial cellular calcium activity after injury between control (HCLE), human primary (HPri), human primary diabetic (HDia), mouse primary (MsPri) and mouse primary diabetic (MsDia) cells. (**A**–**E**) A representative time series shows the wound response in cells before and after injury for control (**A**), HPri (**B**), HDia (**C**), MsPri (**D**), and MsDia (**E**). T stands for time in seconds after injury. (**F**–**J**) A representative normalized intensity graph of the control (**F**), HPri (**G**), HDia (**H**), MsPri (**I**), and MsDia (**J**) initial wound response. (**K**–**O**) A representative maximum intensity topographical map of the control (**K**), HPri (**L**), HDia (**M**), MsPri (**N**), and MsDia (**O**) of initial calcium activity of the wound response. These data represent a minimum of 3 independent replicates.

While communication between cells has been documented in primary and epithelial cell lines, it was not until the calcium propagations between cells were computationally analyzed that we found that the initial response has an ordered wave of propagating calcium activity originating from the wound, while the second, longer-lasting late response displayed disorder [1,19,30]. This initial response is characterized by P2Y2 activity, which determines the directionality of the epithelial sheet during healing [1,19,31,32]. In this study, our goal is to analyze the differences in calcium signaling activity between a HCLE control epithelium, human primary corneal epithelium (HPri), human primary diabetic kidney (HDia) epithelium, and murine primary control (MsPri) and diabetic epithelium (MsDia) in response to injury. In human non-diabetic controls, the initial calcium signaling wave originated from the wound and returns to baseline levels of activity after five minutes (Figure 1A,B). In contrast, the dynamic of the response to injury was different in HDia cells (Figure 1C). In these cells, the initial response had decreased levels of activity that were accompanied by a propagation of fewer signaling events from the wound. In the control, the calcium wave propagated from the wound edge to 8 to 10 cells back from the wound, while in the HDia model the calcium wave only propagated 3 to 4 cells back from the wound, or a 50% decrease. This initial calcium signaling wave was also observed in the mouse models, with the mouse non-diabetic control (Figure 1D) displaying a larger and more robust initial response compared to the mouse diabetic (Figure 1E). The mouse non-diabetic initial response was reduced compared to the human controls. The calcium activity of the initial response can be observed within the averaged intensity of identified cells and is represented as a large initial peak of activity following wounding and a gradual return to baseline levels of intensity over time. An internally normalized average intensity graph is displayed in both human control, primary, and diabetic cell cultures and mouse control and diabetic cell cultures (Figure 1F, Figure 1G, Figure 1H, Figure 1I, and Figure 1J, respectively). All graphs show a similar change in intensity and cellular activity.

To determine the localization of the cells that were active during the initial response, we created maximum intensity topographic maps using FIJI/ImageJ and Zen Blue. Peaks of activity are pseudocolored to represent the cells exhibiting calcium signaling events: blue represents minimal to no activity and red/white represents large/max activity/intensity. A representative control maximum intensity topographical map (Figure 1K) of the initial wound response demonstrates that all cells within the image (an average of six to eight cells back from the wound edge) are activated during the initial calcium wave. A similar amount of cellular activation is found within the human primary topographical map (Figure 1L). In contrast, a representative HDia maximum-intensity topographical map (Figure 1M) of the initial wound response displays decreased size and propagation of the calcium wave from the wound edge, with an average of 3 to 4 cells back from the wound being activated by injury. In comparison to the human models, both mouse topographical maps show a decreased level of activity from the wound edge, with the non-diabetic mouse control (Figure 1N) calcium wave only propagating 3–6 cells from the wound edge and the mouse diabetic cells (Figure 1O) only containing activity directly along the wound.

### 3.2. Second Response—Near-Wound Temporal and Spatial Signaling

The late wound response is a repeating pattern of calcium signaling events found in discreet groups of cells near the wound edge that occurs after the termination of the initial wound response [19]. The late response is characterized by the increased probability of cell–cell event propagation and arises due to the signaling activity of P2X7 and Pannexin-1 [19]. Inhibition of P2X7 eliminates calcium signaling during the late wound response and disrupts cellular migration [1,19], indicating that its activity may play an important role in initiating and coordinating cellular motility.

In control HCLE, non-diabetic human primary and mouse primary models, the late wound response is identified by a cyclic increase in intensity over time caused by small groups of 3–6 cells along the wound edge. This is indicative of cell–cell communication, and the propagation of calcium signaling is observed in normalized average intensity graphs.

**Figure 2:**
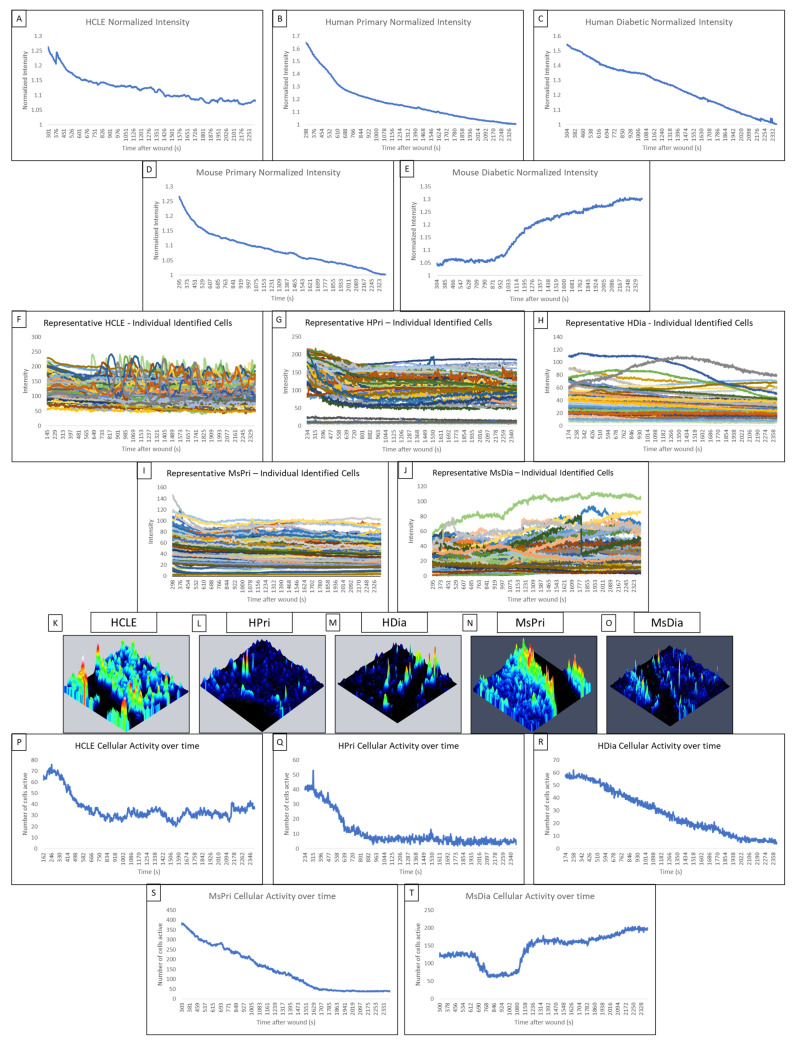
Comparison of the late wound response in control and diabetic cells of human and mouse models. (**A**–**E**) A normalized intensity graph of the HCLE control (**A**), human primary control (**B**), human diabetic (**C**), mouse primary control (**D**), and mouse diabetic (**E**) late calcium wound response. (**F**–**J**) A representative graph of intensity over time for HCLE control (**F**), human primary control (**G**), human diabetic (**H**), mouse primary control (**I**), and mouse diabetic (**J**) cells during the late wound response. (**K**–**O**) A representative maximum-intensity topographical map of the late wound response in HCLE control (**K**), human primary control (**L**), human diabetic (**M**), mouse primary control (**N**), and mouse diabetic (**O**) cells. (**P**–**T**) A representative graph of cellular activity over time during the late wound response in HCLE control (**P**), human primary control (**Q**), human diabetic (**R**), mouse primary control (**S**), and mouse diabetic (**T**) cells. Cells were determined to be active if their intensity exceeded an experimentally determined threshold [14]. All images were collected at a rate of 1 Frame/3 s. These data represent a minimum of 3 independent replicates.

In non-diabetic HCLE controls, increased signaling activity occurs at regular intervals of approximately 5 min throughout the late wound response (Figure 2A). The human primary control also displayed regular increases in intensity during the late wound response (Figure 2B). In contrast, we were not able to detect this periodicity within the HDia cells (Figure 2C). The mouse models mirror the human models, with the non-diabetic control (Figure 1D) displaying several regular-intensity increases compared to the mouse diabetic models (Figure 2E), which did not display a regular pattern of intensity changes. Intensities of individual HCLE and human primary cells after injury (Figure 2F,G) were used to calculate the mean signaling intensity at each time point to determine periodicity. Initiation and propagation of signaling events can be observed multiple times within the same groups of cells. Discreet repetitive patterns of event initiation and propagation, and overall signaling periodicity, are absent in human diabetic cells (Figure 2H). The repetitive patterns of activity were observed in the mouse non-diabetic controls (Figure 2I) but not in the mouse diabetic cells (Figure 2J).

In non-diabetic control cells, there are significantly more signaling events and propagated signaling events within two cells of the wound than when there are three or more cells away. We hypothesize that the wound response in diabetic cells is more spatially limited than in non-diabetic controls. Topographical mapping of signal intensity was used to identify the location of signaling events in the late wound response. In human non-diabetic controls, HCLE, and human primary cells, the majority of cellular activity was localized to groups of high-intensity cells near the wound (Figure 2K,L). HDia topographic maps (Figure 2M) reveal decreased activity near the wound edge with minimal groups of signaling cells. In mouse non-diabetic controls, the activity was also localized along the wound in groups of high-intensity cells (Figure 2N). MsDia topographic maps (Figure 2O) revealed decreased overall activity compared to mouse control.

**Figure 3:**
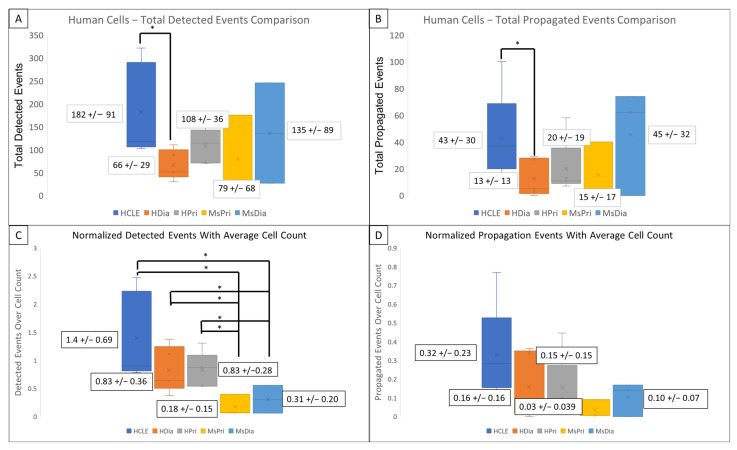
Detected events and Activity Propagation between control and diabetic cells from human and mouse models (**A**–**D**) There were significantly fewer detected signaling events (**A**) and propagated events (**B**) in human diabetic cells compared to human non-diabetic controls (* *p* < 0.05). (**C**,**D**) Events were normalized using the average identified cells from each model. There were significant decreased detected events per cell (**C**) in mouse models compared to human with no significant changes the propagated events per cell (**D**) in mouse models compared to human. These data represent a minimum of 3 independent replicates.

HDia cells display a significant decrease in activity during the late wound response compared to non-diabetic controls. Graphing the number of cells active at each time point reveals similar periodicity to that seen in graphs of cellular intensity in non-diabetic cells, HCLE, and HPri (Figure 2P,Q), but a steady decrease in active cells in HDia cells (Figure 2R). Mouse non-diabetic controls (Figure 2S) reveal a similar periodicity and activity pattern as the human controls, compared to diabetic mouse cells (Figure 2T), which have variability in active cells but maintains a stable number of cells not indicative of cellular activity with a variable intensity. There is a significant decrease in detected events (*p* < 0.05) (Figure 3A) and propagated events (*p* < 0.05) (Figure 3B) between human non-diabetic controls and HDia cells during the late wound response. A normalized analysis of detected events (Figure 3C) and propagated events (Figure 3D) controlling for the amount of cells identified within each cell culture models revealed significant differences within the detected events (*p* < 0.05) between all the human and mouse models but not within the propagation of events.

We hypothesize that as P2X7 signaling is known to generate the late wound response in healthy controls, stimulation of diabetic cells with agonists to P2X7 would generate similar aberrations to those observed in the diabetic late wound response. These experiments allow us to isolate the effects of P2X7 signaling and assess whether it is significantly different in diabetic cells. Experiments were conducted with BzATP, a P2X7 agonist, ATP, and ARL 67156, an inhibitor of ectonucleotidase activity. If the altered signaling profiles in HDia cells were due to decreased or altered ATP levels in the wound bed, the introduction of BzATP and ATP should result in calcium activity similar to non-diabetic controls.

**Figure 4:**
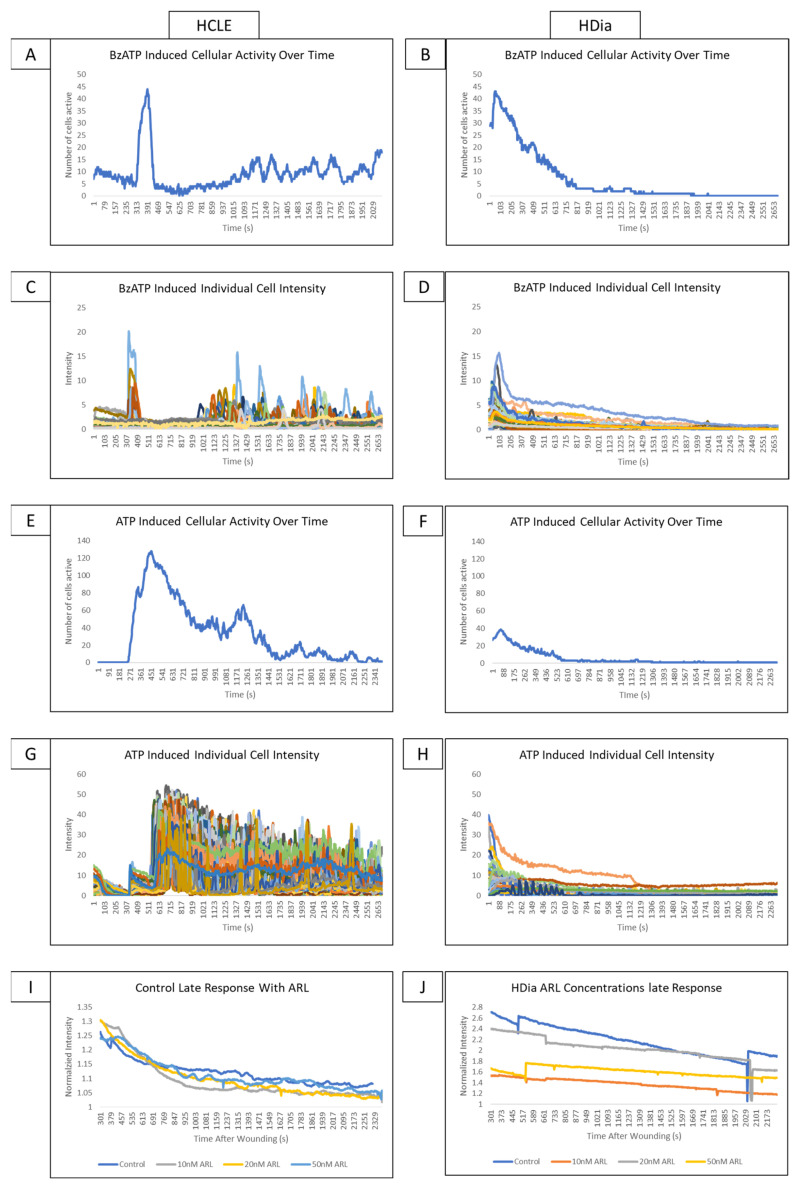
Addressing the role P2X7 activity on control and diabetic signaling profiles using agonists and antagonists. (**A**,**B**) A representative graph of cellular activity over time when HCLE control (**A**) and HDia (**B**) cells are stimulated with the P2X7 agonist BzATP. (**C**,**D**) A representative graph of intensity over time when HCLE control (**C**) and HDia (**D**) cells are stimulated with the P2X7 agonist BzATP. (**E**,**F**) A representative graph of cellular activity over time when HCLE control (**E**) and HDia (**F**) cells are stimulated with ATP. (**G**,**H**) A representative graph of intensity over time when HCLE control (**E**) and HDia (**F**) cells are stimulated with ATP. (**I**,**J**) A normalized average intensity over time graph of the late wound response in control (**I**) and HDia (**J**) with ARL 67156 concentrations: 10 nM, 20 nM, 50 nM. All images were collected at a rate of 1 Frame/3 s. These data represent a minimum of 3 independent replicates.

Stimulation of non-diabetic control cells with BzATP induced a cyclic pattern of cellular activity (Figure 4A) similar to the late wound response. In comparison, stimulation of HDia cells with BzATP (Figure 4B) induced a brief period of increased cellular activity and a rapid return to baseline similar to the activity in the diabetic late wound response. BzATP stimulation in non-diabetic controls induced a cyclic pattern of activity in discreet groups of cells (Figure 4C). The HDia cells displayed cyclic increases and decreases in intensity in individual cells, but these peaks were not part of a larger signaling group (Figure 4D).

When non-diabetic controls are stimulated with ATP, there is an initial spike in calcium signaling activity in all cells that degrades over time, followed by a cyclic pattern of activity that tapers off after approximately 45 min (Figure 4E). In contrast, when HDia cells are stimulated with ATP, there is an initial spike in cellular activity followed only by a return to baseline (Figure 4F). These patterns are also seen when the intensity of each individual control (Figure 4G) and HDia (Figure 4H) cell is graphed over time.

In the non-diabetic controls, inhibition of ectonucleotidase with ARL 67156 does not produce significant changes in detected calcium signaling events and does not alter signal periodicity in the late wound response at any concentration (Figure 4I). This finding is also true in HDia cells (Figure 4J).

### 3.3. Protein Changes Determined Using PLA and SDS PAGE Electrophoresis

**Figure 5:**
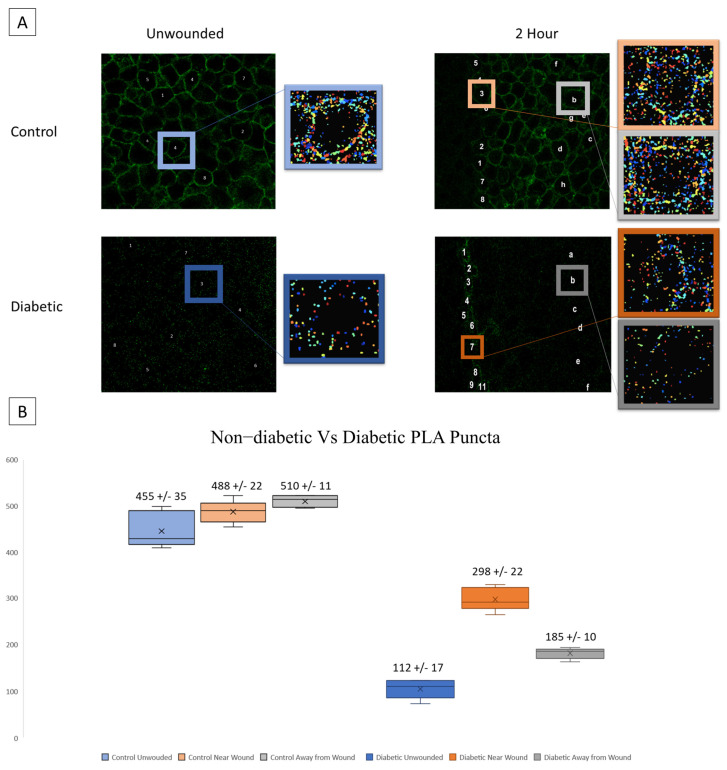
Co-localization of P2X7 and Pannexin-1 after injury is assessed in diabetic murine globes. (**A**) Representative images and identified puncta are shown in both wounded and unwounded control and diabetic tissue. Letters and numbers are used to represent which cells were sampled for analysis. In wounded images, numbers represent cells along the wound edge and letters represent cells back from the wound. Colors correspond to localization (blue is unwounded, orange is near the wound edge, and gray is back from the wound edge; data from diabetic tissues are displayed in a darker color scheme). (**B**) Mean and standard error of the mean of the number of puncta in each condition are displayed in (**B**). Mean ± SEM are plotted, and two-way ANOVA with Tukey’s multiple comparison of means was performed to compare non-diabetic tissue to diabetic.

A possible source of the changes in signaling patterns observed in diabetic cells could be altered interaction or expression of the receptors responsible for the late wound response. We hypothesize that diabetic cells will exhibit significantly increased P2X7 expression yet decreased interactions with Pannexin-1. Proximity Ligation Assay (PLA) was performed to assess the co-localization of P2X7 and Pannexin-1 after injury in diabetic and heathy murine globes [20,33,34,35]. Co-localization puncta were identified using CellProfiler. In previous studies, significant differences were found in P2X7-Pannexin-1 co-localization 2 h after injury in Diet-Induced Obesity (DiO) murine globes compared to 10-week healthy controls but not before injury [20]. We examined this co-localization after injury using NONcNZO10/LtJ diabetic murine globes and compared the findings to controls [20]. Representative images and identified puncta are shown for both wounded and unwounded conditions in control and diabetic tissues. In injury conditions, cells are sampled both at the wound and back from the wound (Figure 5A). Diabetic globes exhibited significantly fewer puncta than controls in both wounded and unwounded conditions. Both diabetics and controls exhibited a significant increase in co-localization after injury both at and back from the wound (*p* < 0.005) (Figure 5B).

SDS PAGE Electrophoresis experiments were performed on cell culture models to determine diabetic P2X7 expression post injury, as previous studies have found altered levels of the purinergic receptor in human diabetic and murine prediabetic models [11,21]. Cells were lysed, and lysate was collected at time points pre- and post-injury (0, 5, 30, and 120 min). Gels were run with equivalent protein and immunoblots were probed using antibodies targeting either the extracellular or the intracellular domain of P2X7.

**Figure 6:**
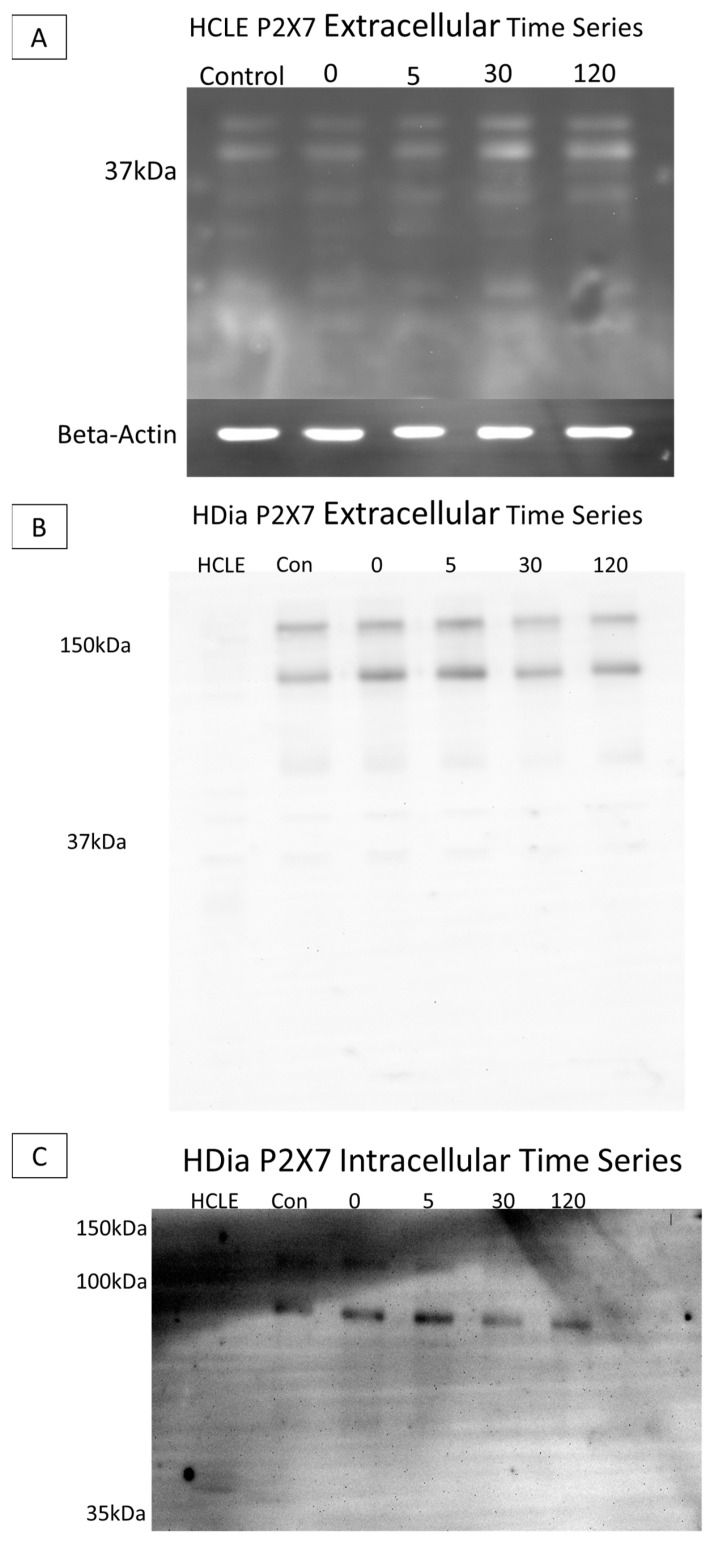
SDS electrophoresis Western blot and membrane P2X7 analysis in control and diabetic cells post injury. (**A**) A representative control HCLE P2X7 receptor extracellular domain time series immunoblot. The ladder has been removed. Column order: unwounded control, immediately after wounding, five minutes after wounding, thirty minutes after wounding, two hours after wounding. Bands were detected at 37 kDa and 24 kDa. Beta-Actin load control is shown below the image. (**B**) A representative human diabetic P2X7 Receptor extracellular domain time series immunoblot. The ladder has been removed. Column order: HCLE control, HDia unwounded control, immediately after wounding, five minutes after wounding, thirty minutes after wounding, two hours after wounding. Bands were detected at 176 kDa and 127 kDa, 68 kDa, 42 kDa, 33 kDa, and 18 kDa. (**C**) A representative human diabetic P2X7 receptor intracellular domain time series immunoblot. The ladder has been removed. Column order: HCLE control, unwounded control, immediately after wound, five minutes after wounding, thirty minutes after wounding, two hours after wounding. Bands were detected at 121kDa, 92 kDa, and 54 kDa.

When non-diabetic control lysates were probed with the antibody to the extracellular domain, P2X7 was detected at 37 kA with no substantial change over time (Figure 6A). When the brightness and contrast were altered (Figure 5B HCLE lane) the 127 and 175 kDa forms were present. In contrast, in the HDia lysates, P2X7 was detected at 175 kDa and 127 kDa. Additional breakdown products were detected at 68 kDa, 42 kDa, 33 kDa, and 17 kDa. The presence of the two higher-molecular-mass species is indicative that a full-length protein is expressed (Figure 6B). In HCLE non-diabetic control intracellular domain P2X7 probes, protein was not detected, potentially indicating a loss of the epitope domain. In contrast, in HDia cells probed with the antibody for the P2X7 intracellular domain, bands were detected at 121 kDa, 92 kDa, and 54 kDa (Figure 6C).

### 3.4. Changes That Occur in Diabetic Corneal Epithelium

Based on the findings observed in cell culture, we hypothesize that there will be significantly less calcium signaling activity in murine globes from a diabetic model compared to a non-diabetic control. To determine if there is a change in cell communication in corneas from diabetic mice, wounding was performed on intact murine globes from non-diabetic control and diabetic mouse models [1,2]. Intact globes were immediately imaged for 1 h using a Zeiss 880 confocal microscope with the 20× air objective. Globes were imaged at several z-planes to observe cellular calcium activity in apical, wing, and basal layers. Images were stabilized using NoRMCorre and cells were manually identified using Regions of Interest (RoI) in FIJI/ImageJ.

**Figure 7:**
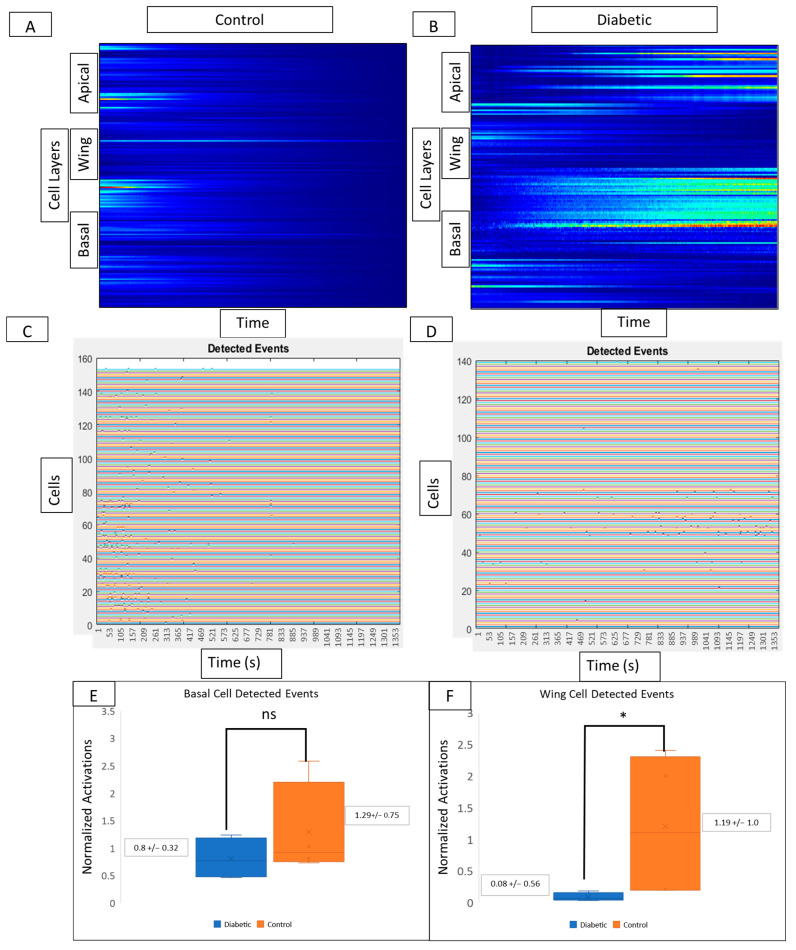
Comparison of cell signaling profiles from ex vivo murine corneal epithelium. Computational analysis of the post-injury wound response at different cellular layers reveals layer-specific differences between diabetic and non-diabetic tissue. (**A**,**B**) Representative MATLAB-generated kymographs in control (**A**) and diabetic (**B**) tissue reveal signaling profiles and event initiation in apical, wing, and basal layers in both control and diabetic tissue. (**C**,**D**) Representative detected events graphs corresponding to MATLAB-generated kymographs in control (**C**) and diabetic (**D**) tissue reveal event initiation in apical, wing, and basal layers in both control and diabetic tissue. Experiments were run for 60 min. Identified cells are represented along the y-axis and the x-axis represents time. There are no significant differences in the number of detected events in the basal cell layer (**E**) between diabetic globes and controls, but there are significantly more signaling events in wing cells from control globes (**F**) (* *p* < 0.05).

Kymograph heatmaps and detected events graphs from both conditions are used to compare activity from different layers of the corneal epithelium. In controls, signaling activity (Figure 7A) and detected events (Figure 7C) are present in wing and basal but not in apical layers. Kymograph and detected event graphs (Figure 7B and Figure 7D, respectively) for diabetic tissue reveal no changes in basal cell activity but decreased activity in wing cells. In the diabetic cornea, there is no significant change in basal cell activity (Figure 7E) and a significant decrease in wing cell activity (Figure 7F) when compared to controls (*p* < 0.05).

**Figure 8:**
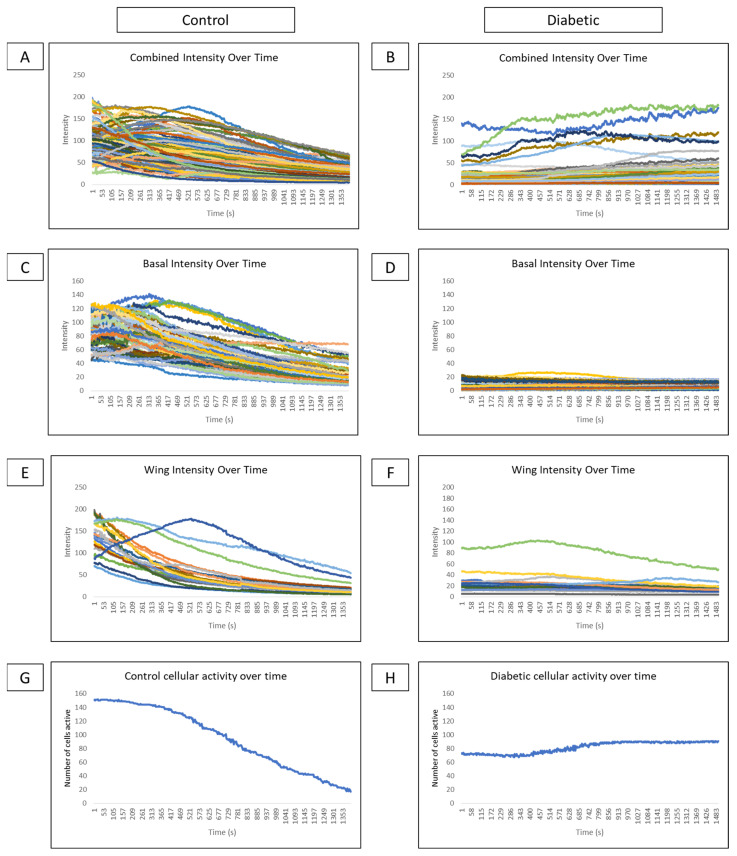
Comparison of intensity and cellular activity over time in control and diabetic ex vivo globes. (**A**–**F**) Representative graphs of cellular intensity over time overall and in different layers in control and diabetic globes. Experiments were run for 60 min. (**A**,**B**) A representative graph of the intensity of all cell layers in control (**A**) and diabetic (**B**) globes. (**C**,**D**) A representative graph of the intensity of basal cell layers in control (**C**) and diabetic (**D**) globes. (**E**,**F**) A representative graph of the intensity of wing cell layers in control (**E**) and diabetic (**F**) globes. (**G**,**H**) A representative graph of cellular activity over time in basal and wing layers from control (**G**) and diabetic (**H**) globes.

Comparisons of cellular intensity of all epithelial layers of a globe over time reveal that control intensity (Figure 8A) is consistently higher than diabetic intensity (Figure 8B). Subdividing intensity based on cell type reveals that basal cell intensity fluctuates over time in several cells in control (Figure 8C) and diabetic (Figure 8D) conditions, indicating that cellular signaling behavior is present. However, there is lower intensity and fewer signaling events in the diabetic globe. In controls, distinct cell signaling events, represented as peaks in intensity, are observed in the wing cell layer (Figure 8E). These signaling peaks are not observed in wing cells of diabetic globes (Figure 8F). Graphing the number of cells active at each time point from both wing and basal layers in control (Figure 8G) and diabetic models (Figure 8H) show high activity after the wound that gradually tapers off in control and minimal changes in cellular activity over time in the diabetic globe.

## 4. Discussion

Previous studies by our lab have focused on changes in wound healing in healthy, aged, and prediabetic models. For example, with age, there was an increase in stiffness yet a decrease in calcium signaling in the murine corneal epithelium [1]. Proximity ligation assay revealed association of P2X7 and Pannexin-1 in unwounded obese and control corneal epithelium. Interestingly, there was an inverse relationship after injury, with an increase in control and decrease in obesity [1,20]. These led us to examine fundamental features of signaling and cell–cell communication between diabetic and control models.

### 4.1. Purinergic Receptors and Potential Effects on the Calcium Activity of the Wound Response

While a number of purinergic receptors are expressed in the corneal epithelium, we have identified specific roles in response to injury for two: P2Y2 and P2X7 [21,36,37,38]. The G-protein coupled receptor, P2Y2, is necessary for induction of the initial calcium propagation, and when in the presence of inhibitors, directionality of wound repair is lost [1,19]. The ionotropic receptor, P2X7, is required for the generation of the later response, and in its absence cellular migration is diminished [1].

A study of the initial calcium propagation following injury in diabetic and control cells did not reveal any major differences. The response is an on–off signal with cells responding but not generating further communication. However, once the initial response returned to baseline and the late wound response began, we observed a number of changes between diabetic and control model systems. In controls, there are typically several discreet groups of cells along the wound edge that exhibit a repeating pattern of calcium propagation in a highly ordered manner. Although individual diabetic cells exhibit signaling behavior, albeit less frequently and at a lower intensity, we did not observe the formation of these signaling groups. When compared to non-diabetic controls, diabetic cells exhibit significantly fewer signaling events and event propagations. Moreover, to understand the changes in the calcium wave propagation that occur in ex vivo diabetic tissue, a quantitative analysis that was recently developed was employed.

Studies have compared the rate of ATP synthesis in age-matched diabetic and non-diabetic patients and reveal that the rate of ATP synthesis is 27% lower in diabetic patients. Unlike in controls, this rate does not increase with insulin stimulation [39,40]. This led us to hypothesize that a potential cause of diminished cellular signaling in diabetic models could be a decrease in the amount of ATP released from injured cells, resulting in a lower concentration of ATP in the wound bed than is typically seen in controls. We observe that the initial signaling wave did not propagate as far from the wound in diabetic cells. However, stimulation of diabetic cells by the agonists BzATP or ATP creates a pattern of calcium activity that aligns with the diabetic wound response as opposed to the more robust response expected if our hypothesis was true. This suggests that ATP concentration in the wound bed is not the main factor producing changes in cellular signaling behavior in diabetic models.

Another potential etiology for the diminished signaling response in diabetic cells is a change in interaction between P2X7 and Pannexin-1. Both proteins promote migration into the wound bed and cell–cell communication during the late wound response. Inhibition of either causes cells to remain near their location of origin instead of migrating into the wound bed [1,12,19]. Inhibition of P2X7 alters both actin and focal adhesion turnover at the leading edge of cultured epithelium after injury [4]. Furthermore, interactions between P2X7 and Pannexin-1, either through paracrine mechanisms or through physical formation of a channel, are well-established in the literature in many organ systems [41,42,43,44,45,46]. In the corneal epithelium of obese mice, co-localization of P2X7 and Pannexin-1 is altered after injury [20]. Although previous results in DiO mice showed co-localization decreasing both at and back from the wound after injury, our findings in diabetic NONcNZO/LtJ mice show an increase in co-localization, which is more characteristic of the changes seen in non-diabetic controls. This finding is interesting because it suggests that cellular signaling profiles in ex vivo murine globes are altered very early in the disease process, even before changes to protein expression and interactions begin. At 9 weeks, the mice are unlikely to have developed an advanced diabetic phenotype, as only 56% of male NONcNZO/LtJ mice develop diabetes by 24 weeks of age [47,48,49,50,51]. This is supported by H&E staining of NONcNZO/LtJ murine pancreases, which reveal hypertrophied instead of atrophied beta islet cells and may explain why the PLA results were not substantially different from controls. This is in contrast to the DiO mice studied previously, which can develop insulin resistance within 1 week of high-fat diet initiation and exhibit a significant decrease in the density of the sub-basal nerve plexus in the cornea by 15 weeks post diet initiation [11,51,52].

### 4.2. Potential Significance of the Altered Calcium Activity Events and Signal Propagation Found within Diabetic Models

The altered cellular activity detected during the wound response in diabetic models underlies changes in either cell recruitment during the wound response or cell–cell communication. Interestingly, the change appears to be a difference in signaling in the wing cells and not the basal cells. These may be generated by underlying changes in the protein cascade.

In the early wound response of all diabetic models, the calcium signaling response is more spatially limited than it is in non-diabetic controls. We speculate that this may be due to aberrations in the mechanisms that differentiate cellular signaling behaviors in age and disease, leading to decreased activation or priming of cells back from the wound, and ultimately to disorganized, uncoordinated, or absent signaling and migration. In other studies, similar behavior was observed in control corneal cells upon inhibition of P2Y2 [1]. Furthermore, phosphoproteomic studies where P2Y2 was inhibited revealed changes in phosphorylation of specific residues on a number of proteins, including paxillin and EGF receptor, indicating a connection between P2Y2 activity and cytoskeletal rearrangement [32,53,54,55,56,57].

In intact globes from diabetic mice, we observed a decrease in both detected events and in overall cellular activity in basal and wing cells compared to non-diabetic mice. This suggests that there is decreased cell–cell communication between layers of the cornea with disease. We speculate that a change in the integrity and regulation of cell–cell junctions as the basal cells mature and take on a wing cell phenotype may affect communication between these layers [58]. The decreased communication between wing cells may also depend on changes in the junctions. In addition, Stepp’s group has demonstrated the epithelial–nerve interactions in dry eye and a number of other insults using detailed morphological studies [59,60]. While we have not examined the nerve structure in the NO mice, there is the possibility that changes in density could alter the activity. In addition, the decreased propagation of events and decreased recruitment of cells during the late wound response may be a critical factor leading to poor wound healing outcomes in the corneal epithelium [61,62,63,64,65,66,67,68].

### 4.3. Future Avenues of Study and Experimentation

These observations lead to many interesting future directions, as we hypothesize that deviation from controls will be observed in ex vivo globes from older NONcNZO/LtJ mice that exhibit a more advanced disease phenotype. PLA experiments on aged NONcNZO/LtJ will allow us to observe how co-localization of P2X7 and Pannexin-1 at the wound changes with disease progression. These will be combined with a study of the junctions. Live cell imaging experiments and computational analysis of aged and diabetic mice may reveal why the signaling behavior in basal and wing cells is impaired.

## 5. Conclusions

In conclusion, there are several significant changes to the wound healing response in diabetic models of the corneal epithelium. In cell culture models, diabetic cells exhibit fewer signaling events overall and fewer signaling events that propagate between neighboring cells when compared to non-diabetic controls. In globes from diabetic mice, there is decreased signaling activity in wing cells. Changes in P2X7 expression and co-localization with Pannexin-1 have been found within diabetic tissue and cells and may contribute to altered calcium signaling activity. Future studies will involve assessing changes in post-injury signaling behavior, protein expression, and protein co-localization with disease progression.

## Data Availability

The data presented in this study can be made available in a publicly archived data repository upon request.
